# Pre-transplant histology does not improve prediction of 5-year kidney allograft outcomes above and beyond clinical parameters

**DOI:** 10.1080/0886022X.2017.1363778

**Published:** 2017-08-23

**Authors:** C. Traynor, A. Saeed, E. O’Ceallaigh, A. Elbadri, P. O’Kelly, D. G. de Freitas, A. M. Dorman, P. J. Conlon, C. M. O’Seaghdha

**Affiliations:** a Department of Nephrology and Transplantation, Beaumont Hospital, Dublin, Ireland;; b Royal College of Surgeons, Dublin, Ireland;; c Department of Pathology, Beaumont Hospital, Dublin, Ireland

**Keywords:** Kidney transplant, graft survival, dual kidney, transplant biopsy, glomerular filtration rate

## Abstract

Pre-implant kidney biopsy is used to determine suitability of marginal donor kidneys for transplantation. However, there is limited data examining the utility of pre-implant histology in predicting medium term graft outcome. This retrospective study examined kidney transplants over a 10-year period at a single center to determine if pre-implant histology can identify cases of eGFR ≤35 ml/min/1.73m^2^ at 5 year follow up beyond a clinical predictive logistic regression model. We also compared outcomes of dual kidney transplants with standard single kidney transplants. Of 1195 transplants, 171 received a pre-implant kidney biopsy and 15 were dual transplants. There was no significant difference in graft and patient survival rates. Median eGFR was lower in recipients of biopsied kidneys compared with standard kidney transplants (44 vs. 54 ml/min/1.73m^2^, *p* < .001). Median eGFR of dual transplant and standard kidney transplants were similar (58 vs. 54 ml/min/1.73m^2^, *p* = .64). Glomerular sclerosis (*p* = .05) and Karpinski Score (*p* = .03) were significant predictors of eGFR at 5-years in multivariate analysis but did not improve discrimination of eGFR ≤35 ml/min/1.73m^2^ at 5-years beyond a clinical prediction model comprising donor age, donor hypertension and terminal donor creatinine (C-statistic 0.67 vs. 0.66; *p* = .647). Pre-implant histology did not improve prediction of medium-term graft outcomes beyond clinical predictors alone. Allograft function of dual transplant kidneys was similar to standard transplants, suggesting that there is scope to increase utilization of kidneys considered marginal based on histology.

## Introduction

Kidney transplantation remains the best treatment option for End-Stage Kidney Disease (ESKD), and the most cost efficient method of renal replacement therapy [1–3]. However, as the need for viable kidneys for transplantation is rapidly increasing, there is a shortage of donor organs [4]. This is driving an increase in the use of extended criteria donors (ECD) which are defined as: (i) Donors greater than 60 years of age. (ii) Donors older than 50 years of age with any two of the following criteria: hypertension, cerebrovascular cause of death, or terminal serum creatinine (SCr) level >130 µmol/dl [5,6].

ECD kidneys are biopsied at the time of organ retrieval and examined by a renal pathologist for renal pathology such as glomerular sclerosis, interstitial fibrosis, tubular atrophy and vascular change [7,8]. On the basis of the biopsy findings, suitable organs can be identified for transplantation, aiming for comparable graft and patient survival outcomes to transplants from optimal deceased donors. Histology can also be used to determine whether recipients receive a single donor kidney or a dual transplant, also called nephron dosing [9]. However, whether dual kidney transplants have comparable graft and patient survival outcomes to single kidney transplants remains debated [10,11].

Much has been reported on clinical predictors of eGFR and graft function at 1-year follow-up, such as donor and recipient age, acute rejection and delayed graft function [12,13]. Histological factors such as glomerular sclerosis and scoring systems of renal pathology have also been examined as eGFR predictors at 1-year follow-up, with conflicting results [14,15].

To our knowledge, research examining histological predictors and composite clinic and pathological predictors of eGFR at 5 years has been limited to date. We previously presented our findings in abstract form [16]. The aim of this study was to determine if baseline donor histology predicts graft outcome at 5 years and whether donor histology adds incremental data to current clinical parameters. In addition, we compared graft outcomes of marginal single and dual kidney transplants to standard deceased donor kidney transplant.

## Methods

### Patient cohort

The study population comprised 1195 kidney transplant recipients who were transplanted between 1997 and 2006 at Beaumont Hospital, Ireland. Minimum clinical follow up time was 5 years post-transplant.

### Predictors

All pre-implant biopsies were wedge biopsies. Each biopsy was graded twice, two weeks apart, by the same consultant renal pathologist. Biopsies were graded using a number of variables including number of sclerosed glomeruli and interstitial fibrosis. Banff’97 classification was also used and measured interstitial fibrosis (ci), tubular atrophy (ct), vascular fibrous intimal thickening (cv) and arteriolar hyaline thickening (ah) on a scale of 0–3.

Using the Banff’97 biopsy scores along with graft glomerular sclerosis, we calculated each patient’s corresponding Karpinksi Score. This is made up of a glomerular, interstitial, tubular and vascular score of 0–3 with a potential sum of 12, with a higher score indicating more advanced changes and a worse prognosis [15]. It should be noted that our study slightly modified the Karpinski score as our histological assessment was based on the Banff’97 biopsy classification. Interstitial fibrosis  < 25% receives a score of 1 using Banff’97 classification whereas the Karpinski score assigns a score of 1 for interstitial fibrosis  < 20%. Therefore, a score of 25% interstitial fibrosis was given a score of 1 rather than 2.

Donor characteristics included age, gender, cause of death as well as terminal serum creatinine, a history of antihypertensive medication use or oral hypoglycemic agents and cold ischemia time. Recipient characteristics included age at transplant, gender, time to graft failure or death if applicable, 1 and 5-year serum creatinine levels, tacrolimus use, human leukocyte antigen mismatches (HLAmm) and panel reactive antibodies (PRA) level.

### Outcome

This study compared the patient and graft survival between three subgroups; standard transplant (1024 non-biopsied single kidney transplants); donor biopsy (156 recipients of single kidney grafts that had a pre-implant biopsy); and Dual Transplants (15 recipients of double kidney transplant who had a pre-implant biopsy). We assessed three outcomes: median eGFR was compared between the three subgroups.; eGFR was then classified as a categorical variable into two groups; high (>35) or low (≤35 ml/min/1.73m^2^) and univariate and multivariate analysis was undertaken to identify clinical and histological predictors of high versus low eGFR; histological parameters were then evaluated against clinical factors using a ROC model. In addition, we examined the histologic characteristics of discarded kidneys that were not transplanted due to histologic findings at the time of the donor biopsy.

### Statistical analysis

Estimated glomerular filtration rate was calculated using the modification of diet in renal disease (MDRD) formula. To be included in eGFR statistical analysis, patients and grafts had to have survived to the 5-year follow up point. Donor and recipient characteristics were measured as continuous and categorical variables. Continuous variables were compared using t-tests. Categorical variables were compared using Pearson Chi squared tests. Histological features were included in multivariate analysis to predict low (≤35 ml/min) or high (>35 ml/min) eGFR. Kaplan–Meier curves were used to compare patient and graft survival. Median eGFR across our three groups was compared using the Kruskal–Wallis test. Logistic regression was used in predictive modeling with subsequent sensitivity and specificity receiver operator curves (ROC) generated. The C statistic derived from the ROC curves was used to determine the extent to which clinical and histology variables discriminate poor graft outcome, defined as an eGFR ≤ 35 ml/min. Stata version 10 (College Station, TX) was used for data analysis. A *p* value < .05 was deemed to be significant. All data were obtained from the Irish Kidney Transplant Registry which incorporates all data on kidney transplant recipients in the Republic of Ireland.

## Results

### Population demographics

During the study period, there were a total of 1195 kidney transplants performed of which 171 underwent pre-implant biopsy. Of these, 156 received a single kidney transplant and 15 received a dual kidney transplant. The characteristics of these groups are summarized in Table 1. Kidneys selected for pre-implant biopsy tended to be from older donors, female donors and have a non-traumatic cause of death. Recipient factors were older age at time of transplant and tacrolimus use (versus cyclosporine) post-transplant.

**Table 1.** Patient demographics.

Characteristics	Non-biopsied (*n* = 1021)	Biopsied (*n* = 171)	*p* Value
Donor age^a^ (years)	34.4 ± 14.1	52.0 ± 9.9	<.001
Donor sex (male %)	58%	49%	.02
Donor cause of death %			
Trauma	46.9%	29.8%	<.001
Non trauma	53.1%	70.2%	–
Cold ischemia time (h)^a^	20.5 ± 5.6	19.2 ± 4.6	.475
Number of HLA mismatches^a^	2.96 ± 1.34	3.09 ± 1.32	.115
PRA GROUP %			
1 (<10%)	77.4%	81.3%	.251
2 (10–49%)	10.6%	11.1%	–
3 (>50%)	12%	7.6%	–
Recipient age^a^ (years)	42.0 ± 14.2	54.7 ± 12.1	<.001
Recipient sex (male)	62%	66%	.320
Tacrolimus use %	53.8%	77.2%	<.001
Acute rejection %	23.7%	24.0%	.944

a Mean ± standard deviation.

PRA: panel reactive antibody; HLA: human leukocyte antigen.

### Patient and graft survival outcomes

Patient and graft survival curves at 5 years follow-up were examined, comparing rates for the three study groups. Patient survival rates for standard, donor biopsy and dual kidney transplants were 90.0, 85.6 and 78.9%, respectively (*p* = n.s.). Graft survival rates for standard, donor biopsy and dual kidney transplants were 80.0, 76.6 and 79.0%, respectively (*p* = n.s.).

#### Glomerular filtration rate

**Figure 1.**
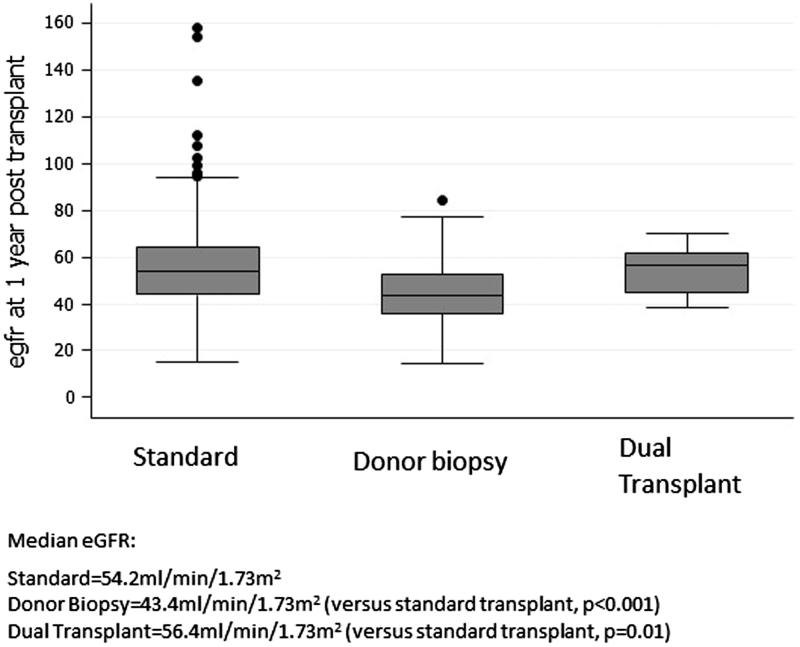
Box plot of median eGFR at 1 year in the three groups.

**Figure 2.**
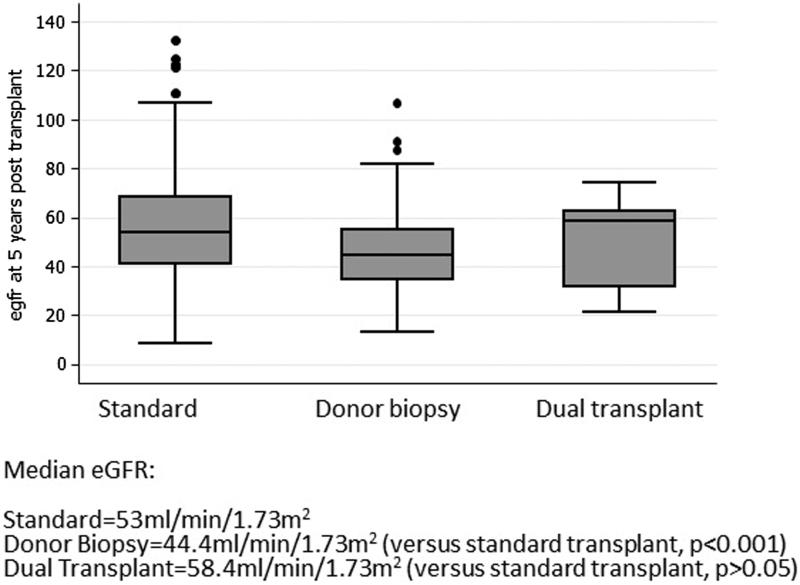
Box plot of median eGFR at 5 years in the three groups.

Median eGFR at 1 year was 54.2, 43.4, and 56.4 ml/min/1.73m^2^ for standard, donor biopsy and dual kidney transplants, respectively (Figure 1). Median eGFR at 5 years was significantly lower in the donor biopsy group than those with a standard transplant (44.4 ml/min vs. 53.9 ml/min, *p* < .001). There was no significant difference in median eGFR at 5 years in the dual kidney group compared to the standard kidney transplant group (58.4 vs. 53.9 ml/min/1.73m^2^, *p* = .6) (Figure 2).

### Predictive modeling

Donor, recipient and histological characteristics were then examined as continuous predictors of eGFR ≤35 ml/min/1.73m^2^ at 5 years (Tables 2 and 3) in patients who had a pre-implant biopsy. Donor age (*p* = .03) and biopsy proven acute rejection (*p* = .04) were significantly associated with eGFR of  < 35 ml/min/1.73m^2^ at 5 years in univariate analysis. On multivariate analysis of histology findings, the percentage glomerular sclerosis (*p* = .045) and Karpinksi score (*p* = .033) both had a significant association with 5 year eGFR, when examined with clinical variables. The other histological factors examined were not significantly associated with eGFR at 5 years (Table 3).

**Table 2.** Donor and recipient characteristics and association with eGFR at 5 years in patients who had a pre-implant biopsy.

Characteristics	eGFR < 35 ml/min	eGFR > 35 ml/min	*p* Value
Donor age (years)^a^	54.2 ± 10.1	51.3 ± 9.2	.035
Terminal creatinine(µmol/l)^a^	87.2 ± 44.0	87.67 ± 38.5	.679
Donor cause of death (%)			
Trauma	29.4%	28.4%	.913
Non trauma	70.6%	71.6%	–
Donor hypertension (%)	33.3%	20.0%	.119
Donor diabetes (%)	3.1%	0.00%	.302
Cold ischemia time (h)^a^	18.7 ± 3.2	19.7 ± 4.4	.484
HLA mismatches^a^	3.1 ± 1.4	3.1 ± 1.3	.591
PRA Group %			
1 (<10%)	88.2%	77.9%	.396
2 (10–49%)	5.9%	13.7%	–
3 (>50%)	5.9%	8.4%	–
Donor Hx CMV disease %	2.94%	6.32%	.456
Recipient age (years)^a^	59.5 ± 10.9	59.9 ± 12.1	.714
Recipient sex (male)	58.8%	70.5%	.211
Tacrolimus use %	73.5%	83.2%	.223
Acute rejection %	35.3%	17.9%	.037

a Mean ± standard deviation.

**Table 3.** Histological parameters using Banff 97 classification (ah, ci, ct, cv and cg) and karpinski score and association with eGFR at 5 years.

Histological parameters	*n*	eGFR <35ml/min	eGFR >35ml/min	*p* Univariate	*p* Multivariate
Glomerular sclerosis					
0%	15	2.9%	14.7%	.074	.045
10%	72	50.0%	57.9%	–	–
10–19%	32	38.2%	20.0%	–	–
>20%	10	8.8%	7.4%	–	–
Interstitial fibrosis					
0–19%	25	18.8%	20.0%	.642	.103
20–29%	59	40.6%	48.4%	–	–
>30%	43	40.6%	31.6%	–	–
Arteriolar hyalinosis (ah)					
0	41	21.2%	36.6%	.379	.182
1	62	57.6%	46.2%	–	–
2	22	21.2%	16.1%	–	–
3	1	0.0%	1.1%	–	–
Chronic interstitium (ci)					
0	3	0.0%	3.2%	.510	.119
1	78	55.9%	62.1%	–	–
2	48	44.1%	34.7%	–	–
Chronic tubules (ct)					
0	8	0.0%	8.4%	.125	.782
1	113	97.1%	84.2%	–	–
2	8	2.9%	7.4%	–	–
Chronic vessels (cv)					
0	45	21.2%	43.7%	.038	.054
1	69	69.7%	52.9%	–	–
2	6	9.1%	3.4%	–	–
Chronic glomerulopathy (cg)^a^					
0	104	76.5%	82.1%	.461	.162
1	25	23.5%	17.9%		
Karpinski^b^					
0–3	30	5.9%	29.8%	.010	.033
4–5	75	70.6%	54.3%		
6–9	23	23.5%	15.9%		

a cg is graded 0–1 using Banff classification; however, there were no biopsies graded >1 in study cohort.

b Karpinski score is graded 0–12; however, there were no biopsies graded >9 in study cohort.

**Figure 3.**
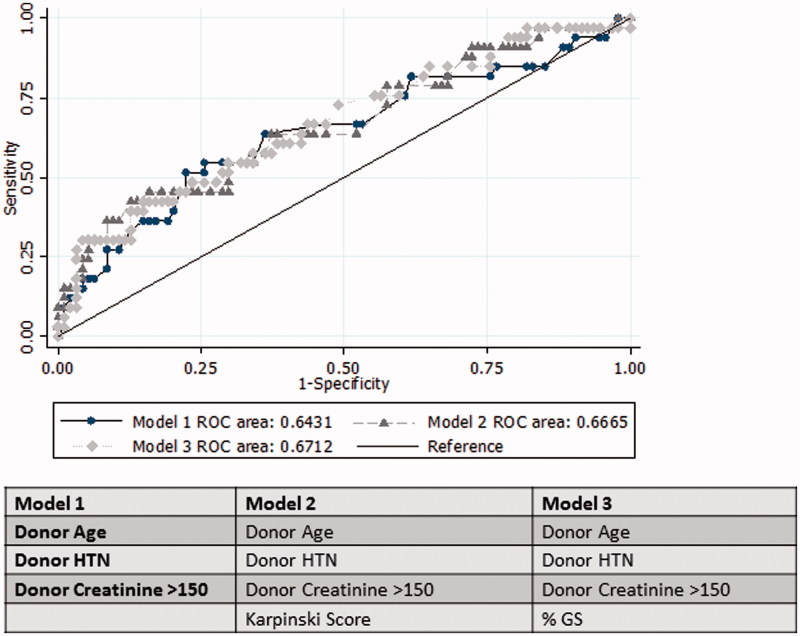
ROC predictive model.

Based on these histological associations, three composite clinic-histological models were generated to determine if histological score improved the C-statistic beyond using clinical factors only, as predictors of eGFR at 5 years (Figure 3) [16]. A clinical predictive model comprising donor age, donor hypertension and terminal donor creatinine >150 μmol/l performed moderately well in predicting 5-year graft function (C-statistic 0.64). Adding in the Karpinski score failed to meaningfully improve on this (C-statistic 0.67, *p* = .65).

### Discarded kidneys

There were 29 kidneys biopsied in 16 potential donors that were not subsequently transplanted due to biopsy findings at procurement. On review of the biopsy results of these patients compared to the biopsied kidneys that were transplanted: the mean glomerular sclerosis was 25%±14.5 vs. 7.9%±7.1 (*p* < .001), the mean interstitial fibrosis was 40%±11.3% vs. 24%±8.1% (*p* < .001) and the mean Karpinski score was 8.2 ± 2 vs. 4.2 ± 1.5 (*p* < .001).

## Discussion

The principal findings of the study are fourfold. First, median eGFR at 5 years follow up was significantly lower in those that required pre-implant biopsies and received a single kidney transplant than those that did not require a biopsy. Second, recipients of dual kidney transplants had similar eGFR at follow-up to those receiving standard single kidney transplants. Third, a clinical predictive model comprising donor age, donor hypertension and terminal donor eGFR performed moderately well in predicting 5-year graft function. Lastly, although glomerular sclerosis and the Karpinski score were associated with graft outcome on multivariate analysis, adding it to a clinical model did not improve prediction of medium-term graft outcomes above clinical data alone. Taken together, these results suggest that histological assessment of the allograft on the day of transplant adds little additional prognostic information after clinical parameters are taken into account.

There have been several studies examining predictors of eGFR and graft survival at 1-year post transplantation [17–19]. Moore et al. identified clinical predictors incorporated in the Donor Risk Score (donor race; donor history of hypertension/diabetes/death due to CVA; cold ischemia time; HLA mismatch; donor/recipient CMV match) as having the strongest association with creatinine at 1 year [17]. Hall et al found no association between pre-implant biopsy acute tubular necrosis and either delayed graft function or graft failure [18]. A study by Anglicheau et al. addressed the effects of clinical and histological parameters on eGFR at 1-year post transplant and found glomerular sclerosis and arteriolar hyalinosis to be significant predictors of eGFR  < 25 ml/min [19]. They also found a composite scoring system including donor creatinine >150 μmol/l, donor hypertension and glomerular sclerosis >10% to be the strongest predictor of low eGFR at 1 year.

There is limited data on the impact of histology on 5-year graft function. The early identification of kidney transplant recipients at higher risk for subsequent transplant dysfunction has clinical implications with regards to patient management and follow-up. Consistent with the findings by Anglicheau et al., our study found glomerular sclerosis to be a significant predictor of low eGFR (≤35 ml/min) on multivariate analysis. Karpinski score was also a significant predictor of 5-year eGFR on both univariate and multivariate analysis. However, once we added glomerular sclerosis and Karpinski score to a predictive model incorporating clinical variables readily available at the time of organ procurement; they did not provide additional predictive value above the clinical factors alone.

There was no significant difference in patient and graft survival between the three groups of patients. The 5-year graft survival rates for standard, donor biopsy and dual kidney transplants of 80, 77 and 79%, respectively, suggest reasonable outcomes for many patients who would otherwise have remained on the transplant waiting list. Pre-implant biopsy of marginal donors appropriately selected kidneys that were suitable for transplant. As expected with marginal donors, recipient graft function was reduced with a significantly lower eGFR in those that required pre-implant biopsies and received a single kidney transplant than those with a standard transplant (44.4 ml/min vs. 53.9 ml/min) (*p* < .001). Recipients of dual kidneys had an eGFR of 58.4 ml/min; however, this was non-significant given the small number of patients. It has been suggested that careful selection of kidneys based on clinical and histological criteria would allow the use of kidneys that would not otherwise have been used for transplantation [20]. The results of our study support this.

There were 29 kidneys discarded on the basis of adverse histology and it is unclear what level of kidney function could be achieved if these kidneys were utilized. Pre-implant biopsy resulted in successful designation of kidneys into single transplants and dual transplants. However, consideration should be given to transplanting kidneys with higher chronicity scores that would otherwise be discarded with an expectation of acceptable graft outcome.

As biopsy grading was carried out by the same renal pathologist, inter-observer variability of the biopsy findings was avoided. Limitations to our study include the fact our study was a single center, retrospective design with a predominantly white population therefore our results may not be applicable to other study populations. Another potential limitation was selection bias, as kidneys from older donors may have been excluded from transplantation by the surgeons before biopsy, however, this reflects real world practice.

In conclusion, a predictive model comprising donor age, donor hypertension and terminal donor eGFR performed moderately well in predicting 5-year graft function. While a lower Karpinski score and glomerular sclerosis was associated with higher eGFR at 5 years, adding either one of these to the clinical predictive model did not improve the discrimination of graft outcomes. Although 5-year eGFR for marginal kidney recipients was lower than those with standard kidney transplants, graft function was nonetheless substantial and graft survival was similar to standard donors. This encouraging medium term outcome suggests that there is scope to increase the utilization of kidneys considered marginal based on adverse histology.

## References

[1] Wolfe RA, Ashby VB, Milford EL, et al Comparison of mortality in all patients on dialysis, patients on dialysis awaiting transplantation, and recipients of a first cadaveric transplant. N Engl J Med. 1999;341:1725–1730. 10580071 10.1056/NEJM199912023412303

[2] Abecassis M, Bartlett ST, Collins AJ, et al Kidney transplantation as primary therapy for end-stage renal disease: a National Kidney Foundation/Kidney Disease Outcomes Quality Initiative (NKF/KDOQITM) conference. Clin J Am Soc Nephrol. 2008;3:471–480. 18256371 10.2215/CJN.05021107 PMC2390948

[3] de Wit GA, Ramsteijn PG, de Charro FT. Economic evaluation of end stage renal disease treatment. Health Policy. 1998;44:215–232. 10182294 10.1016/s0168-8510(98)00017-7

[4] Xue JL, Ma JZ, Louis TA, et al Forecast of the number of patients with end-stage renal disease in the United States to the year 2010. J Am Soc Nephrol. 2001;12:2753–2758. 11729245 10.1681/ASN.V12122753

[5] Ojo AO. Expanded criteria donors: process and outcomes. Semin Dial. 2005;18:463–468. 16398707 10.1111/j.1525-139X.2005.00090.x

[6] Merion RM, Ashby VB, Wolfe RA, et al Deceased-donor characteristics and the survival benefit of kidney transplantation. JAMA. 2005;294:2726–2733. 16333008 10.1001/jama.294.21.2726

[7] Stratta RJ, Rohr MS, Sundberg AK, et al Increased kidney transplantation utilizing expanded criteria deceased organ donors with results comparable to standard criteria donor transplant. Ann Surg. 2004;239:688–695. 15082973 10.1097/01.sla.0000124296.46712.67 PMC1356277

[8] Racusen LC, Solez K, Colvin RB, et al The Banff 97 working classification of renal allograft pathology. Kidney Int. 1999;55:713–723. 9987096 10.1046/j.1523-1755.1999.00299.x

[9] Remuzzi G, Grinyò J, Ruggenenti P, Double Kidney Transplant Group (DKG), et al Early experience with dual kidney transplantation in adults using expanded donor criteria. J Am Soc Nephrol. 1999;10:2591–2598. 10589699 10.1681/ASN.V10122591

[10] Gandolfini I, Buzio C, Zanelli P, et al The Kidney Donor Profile Index (KDPI) of marginal donors allocated by standardized pretransplant donor biopsy assessment: distribution and association with graft outcomes. Am J Transplant. 2014;14:2515–2525. 25155294 10.1111/ajt.12928 PMC4400114

[11] Rigotti P, Capovilla G, Di Bella C, et al A single-center experience with 200 dual kidney transplantations. Clin Transplant. 2014;28:1433–1440. 25297945 10.1111/ctr.12475

[12] Hariharan S, McBride MA, Cherikh WS, et al Post-transplant renal function in the first year predicts long-term kidney transplant survival. Kidney Int. 2002;62:311–318. 12081593 10.1046/j.1523-1755.2002.00424.x

[13] Resende L, Guerra J, Santana A, et al First year renal function as a predictor of kidney allograft outcome. Transplant Proc. 2009;41:846–848. 19376368 10.1016/j.transproceed.2009.01.066

[14] Gaber LW, Moore LW, Alloway RR, et al Glomerulosclerosis as a determinant of posttransplant function of older donor renal allografts. Transplantation. 1995;60:334–339. 7652761 10.1097/00007890-199508270-00006

[15] Karpinski J, Lajoie G, Cattran D, et al Outcome of kidney transplantation from high-risk donors is determined by both structure and function. Transplantation. 1999;67:1162–1167. 10232568 10.1097/00007890-199904270-00013

[16] Traynor C, Dorman T, de Freitus D, et al Preimplant donor histology does not improve discrimination of 5 year kidney allograft outcomes. Am J Transplant. 2016;16(S3):405–798.

[17] Moore J, Ramakrishna S, Tan K, et al Identification of the optimal donor quality scoring system and measure of early renal function in kidney transplantation. Transplantation. 2009;87:578–586. 19307797 10.1097/TP.0b013e3181949e71

[18] Hall IE, Reese PP, Weng FL, et al Preimplant histologic acute tubular necrosis and allograft outcomes. Clin J Am Soc Nephrol. 2014;9:573–582. 24558049 10.2215/CJN.08270813 PMC3944773

[19] Anglicheau D, Loupy A, Lefaucheur C, et al A simple clinico-histopathological composite scoring system is highly predictive of graft outcomes in marginal donors. Am J Transplant. 2008;8:2325–2334. 18785957 10.1111/j.1600-6143.2008.02394.x

[20] D’Arcy FT, O’Connor KM, Shields W, et al Dual kidney transplantation with organs from extended criteria cadaveric donors. J Urol. 2009;182:1477–1481. 19683744 10.1016/j.juro.2009.06.021

